# The era of bioengineering: how will this affect the next generation of cancer immunotherapy?

**DOI:** 10.1186/s12967-017-1244-2

**Published:** 2017-06-19

**Authors:** Michele Graciotti, Cristiana Berti, Harm-Anton Klok, Lana Kandalaft

**Affiliations:** 1 Center of Experimental Therapeutics, Department of Oncology, University Hospital of Lausanne, Lausanne, Switzerland; 2 Laboratoire des Polymères, Institut des Matériaux and Institut des Sciences et Ingénierie Chimiques, École Polytechnique Fédérale de Lausanne (EPFL), Bâtiment MXD, Station 12, 1015 Lausanne, Switzerland; 3 Ludwig Cancer Research Center, University of Lausanne, Lausanne, Switzerland

## Abstract

**Background:**

Immunotherapy consists of activating the patient’s immune system to fight cancer and has the great potential of preventing future relapses thanks to immunological memory. A great variety of strategies have emerged to harness the immune system against tumors, from the administration of immunomodulatory agents that activate immune cells, to therapeutic vaccines or infusion of previously activated cancer-specific T cells. However, despite great recent progress many difficulties still remain, which prevent the widespread use of immunotherapy. Some of these limitations include: systemic toxicity, weak immune cellular responses or persistence over time and most ultimately costly and time-consuming procedures.

**Main body:**

Synthetic and natural biomaterials hold great potential to address these hurdles providing biocompatible systems capable of targeted local delivery, co-delivery, and controlled and/or sustained release. In this review we discuss some of the bioengineered solutions and approaches developed so far and how biomaterials can be further implemented to help and shape the future of cancer immunotherapy.

**Conclusion:**

The bioengineering strategies here presented constitute a powerful toolkit to develop safe and successful novel cancer immunotherapies.

## Background

**Fig. 1:**
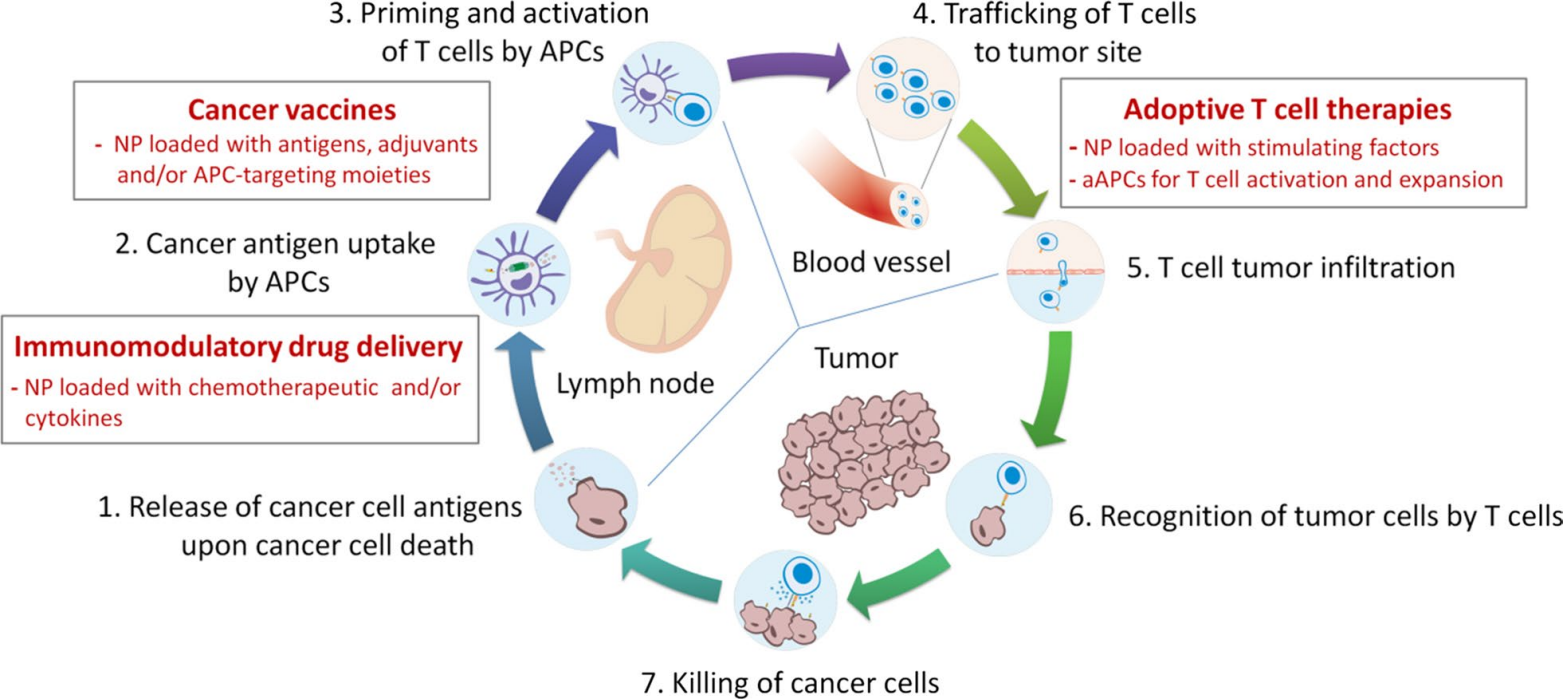
The cancer immunity cycle. Diagram illustrating the seven major steps involved in the generation of an immune response against cancer with main bioengineering approaches developed so far (*in red*). *aAPCs* artificial antigen presenting cells, *APCs* antigen presenting cells, *NPs* nanoparticles. Adapted from [171]

Since its first application in 1890 by William Coley who treated cancer patients with a mixture of killed bacteria observing complete remission in 10% of cases [1], cancer immunotherapy has “travelled” a long way, culminating in 2010 with the first personalized immunotherapy approved by FDA against prostate cancer [2]. However, despite its surprising progress, many hurdles still persist that hamper success rates and wide applicability [3]. An anticancer immune response usually consists of an intricate network of events involving both innate and adaptive immune system first triggered by the uptake, processing and presentation of tumor antigens by antigen presenting cells (APCs), followed by T cell priming and activation and concluding with the infiltration of effector T cells to the tumor site where they exert their cytotoxic activity potentially leading to tumor clearance (Fig. 1). Although this is a spontaneous and natural occurring process, tumors usually develop various mechanisms in order to escape this immune response (e.g. antigen loss, release of immunoinhibitory signals in the tumor microenvironment and others), usually referred to as immunoediting [4]. Several therapeutic approaches acting at different stages of the cancer immunity cascade have been developed over the years to overcome tumor immune escape. These can be classified in two: immunotherapies where cytokines or other immunomodulatory molecules are submitted to patients eliciting a cellular immune response in vivo, or immunotherapies where immune cells are generated, stimulated and expanded ex vivo and then injected into patients. In this review we will describe the current challenges that these approaches present and how biomaterials and bioengineering could help solving central issues to advance and improve cancer immunotherapy.

## Immunomodulatory drug delivery

**Fig. 2:**
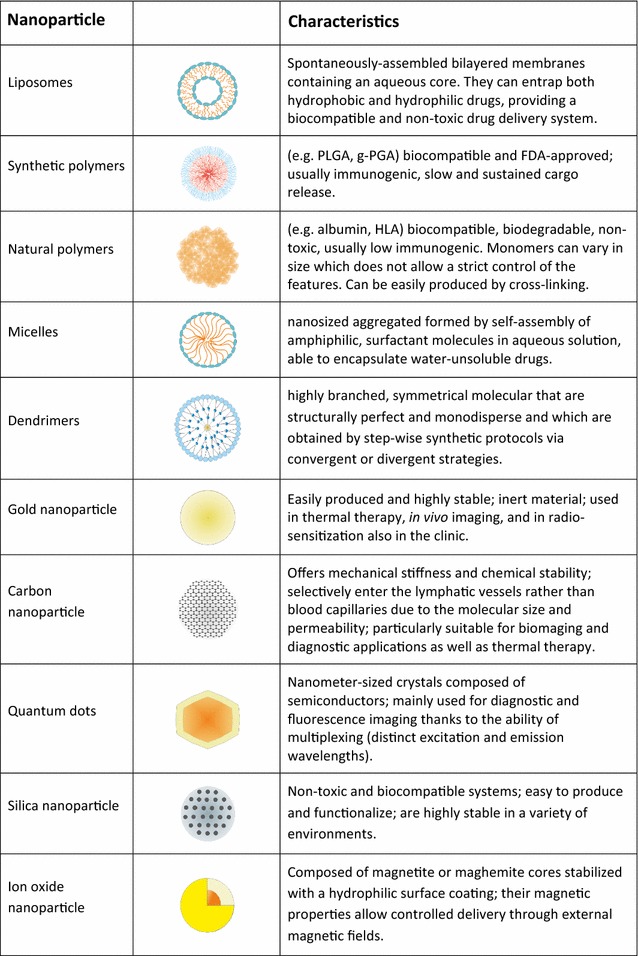
Nanoparticle classification and main characteristics. *γ-PGA* poly(γ-glutamic acid), *HA* hyaluronic acid, *PLGA* poly(lactic-*co*-glycolic acid)

Chemotherapy is one of the most common therapies currently used for cancer treatment, however its application is often limited by large side effects linked to cytotoxic activity also on healthy tissues and cells, especially in patients already compromised by the disease. Although the use of cytotoxic drugs was traditionally thought to be immunosuppressive, this view is currently being changed by raising evidence [5]. One major factor to contribute in this sense is the so-called immunogenic cell death (ICD) which consists of the release of immunostimulatory molecules by cancer cells upon apoptotic cell death, leading to increased antigen uptake by dendritic cells (DCs) and immunization [6]. In recent years, to overcome side effects related to systemic administration, cancer drugs have been encapsulated in nanoparticles such as liposomes or poly(lactic-*co*-glycolic acid) (PLGA) nanoparticles (Fig. 2) and several are now FDA approved or being tested in clinical trials [7]. Nanoparticle encapsulation ensures tumor delivery thanks to both high vascular permeability and poor lymphatic drainage of the diseased tissue, leading to passive accumulation of nanoparticles at the tumor site (so-called EPR effect: enhanced permeability and retention effect) [8]. While the EPR effect has been shown to be effective in rodent models, translating this concept to the treatment of human cancers has proven more difficult [9]. Moreover, nanoparticles also provide increased drug stability due to shielding from the external environment, sustained release over time and increased local concentration. Interestingly, the impact of these approaches in the immunotherapy field is only starting to emerge very recently. A study by Zhao et al. showed for example that delivery of oxaliplatin by PLGA nanocarriers [10] (NP-OXA) induced a stronger immune response both in vitro (in co-culture assays of stimulated DCs and T-cells) and in immunocompetent mice, compared to oxaliplatin alone (OXA). In particular, NP-OXA-treated mice showed a higher proportion of tumor infiltrated lymphocytes (TILs), higher IFN-γ expression and increased tumor shrinkage compared to OXA treatment alone [10]. These results show that encapsulation improved the drug immunogenicity by increasing ICD, thus leading to a more pronounced immune response. On the contrary, no significant differences were recorded between mice treated with gemcitabine alone or encapsulated, confirming that not all chemotherapeutic drugs and formulations are able to induce ICD or possess immunostimulatory effects [11]. To that point, it will be important in the future to extend the test of chemotherapeutic nanomedicines also in immunocompetent mice instead of just the standard immunodeficient mice model [12] in order to investigate a possible role of the immune system in the response and fully reveal therapeutic potentials.

A similar strategy of nanoparticle encapsulation is also currently being pursued for the delivery of cytokines to boost and sustain the immune response against cancer cells in a more direct manner. Cytokines play a crucial role in stimulating and regulating the immune response against antigens, but their use in the clinic has been greatly limited due to harmful side effects linked with their pleiotropic nature and often dual role in simultaneously stimulating and suppressing the immune response at different levels [13]. As for chemotherapeutic drugs, nanoparticle encapsulation offers a potential solution also in this context by providing target delivery at the tumor site, therefore avoiding systemic toxicity. Recently, several groups have tested the delivery of IL-12 loaded on chitosan nanoparticles either as a recombinant protein [14, 15] or as encoding DNA for gene therapy [16], obtaining promising results. IL-12 is a powerful, proinflammatory cytokine that enhances T_H_1 cell differentiation, proliferation of activated T cells and natural killer (NK) cells and cell-mediated immunity [17]. Zaharoff et al. reported that IL-12/chitosan nanoparticles were superior to IL-12 alone in terms of overall survival and cytokine production in a mouse model of bladder cancer, further inducing 100% protection to tumor rechallenge in previously cured mice, lasting lymphocytic infiltration and a tumor-specific adaptive immune response [14, 15]. Significant results in terms of cytokine production and positive therapeutic outcome in mice have also been recorded with nanoparticle-mediated IL-12 gene therapy [16]. In addition, nanoparticle encapsulation has been reported for IL-2 [18–20], IL-15 [21], IL-10 siRNA [22], GM-CSF [23, 24], and the toll-like receptor (TLR) agonists CpG oligodeoxynucleotides (CpG-ODN) [25–27] and Poly I:C [28, 29], with positive outcomes in mouse models (Table 1). All these studies collectively confirmed the previous assumption that nanoparticle formulations are safer and induce better therapeutic effects than their free-soluble counterparts due to controlled local administration and higher concentration at the tumor site in virtue of the EPR effect. This innovative approach opens therefore a new scenario where immunomodulatory agents previously discontinued due to toxicity could be potentially reconsidered, improved by encapsulation and tested for future cancer immunotherapy. On the other hand, also treatments discarded due to low efficacy could be revisited and implemented in new biomaterials formulations [30]. Interestingly, a novel approach combining delivery of both cytotoxic drugs and cytokines through nanoparticles is also being pursued. The rationale here is of a “two-hit” strike to cancer cells: a “first-hit” due to the drug cytotoxic effect leading to cell apoptosis, activation of APCs and subsequent triggering of an immune response, and a “second-hit” which improves and sustains such immune response through the cytokine/TLR agonist action [10]. An example of this approach is the administration of lipid-coated cisplatin nanoparticles (LPC) followed by CpG-encapsulated liposomes 1 day after in a melanoma mouse model. Results showed that the combination treatment was far superior than both single mono-therapies in terms of controlling tumor growth, IFN-γ production, favourable cytokine profile and immunological memory [31]. Another study used a sequential administration of hyaluronic acid-paclitaxel complex followed by two types of PLGA nanoparticles loaded respectively with CpG-ODN and IL-10 siRNA also showing effective and synergistic results [32]. Other studies in a mouse model successfully used instead simultaneous co-delivery of chemotherapeutic drugs and immunomodulatory agents loaded in the same nanoparticle (Table 1) [33, 34].

**Table 1:** List of recent studies investigating nanoparticle-mediated delivery of immunomodulatory agents

Carrier	Agent	Model system	Outcome^a^	References
mPEG-PLGA	Oxaliplatin	Pan02 pancreatic cancer mouse model	Increased TIL levels, increased IFN-γ production	[10]
Chitosan	IL-12	MB49 bladder tumor mouse model	Induced antitumoral activity and T_H_1 cytokine expression	[14]
Chitosan	IL-12	MB49 and MBT-2 bladder tumor mouse models	100% protection to tumor rechallenge in previously cured mice	[15]
Liposome	Cisplatin CpG	B16–F10 melanoma mouse model	Tumor clearance, long-term protection, T_reg_ downregulation	[31]
Nanodiamond	CpG	B16–F0 melanoma and 4T1 breast cancer mouse models	IL-12 production and tumor shrinkage	[25]
PEI	IL-2 plasmid	B16–F1 melanoma mouse model	Reduced tumor growth, prolonged survival, increased TIL tumor infiltration	[18]
Chitosan	IL-2 plasmid	BALB/c mouse inoculated with WEHI-164 in vitro transfected cells	Tumor mass volume decrease	[162]
Hydroxyethyl starch	IL-2	C57BL/6 mouse model; Rag2^−/−^γc^−/−^ mice reconstituted with human CD4^+^ T cells	In vivo T cell specific uptake	[20]
Nanolipogel	IL-2 and TGF-β inhibitor	B16-F10 melanoma mouse model	Increased survival Increased CD8^+^ T cells tumor infiltration	[19]
Polylactic acid	IL-12, IL-18, TNF-α alone or in combinations	4T1 breast cancer mouse models	IL-12 and TNF-α combination was the best condition for controlling tumor growth	[163]
PLGA-PEI	CpG, IL-10 siRNA	A20 B-cell lymphoma mouse model	Improved T_H_1/T_H_2 cytokine expression ratio, Increased survival	[22]
HA PLGA PLGA	Paclitaxel CpG IL-10 siRNA	B16–F10 melanoma mouse model	Tumor growth inhibition High T_H_1/T_H_2 cytokine expression ratio	[32]
PPS	CpG	E.G7-OVA and B16F10 mouse model	Enhanced T_H_1 cytokine secretion and protection to tumor rechallenge	[26]
silica	GM-CSF	In vitro	Increased macrophage proliferation	[24]
Zinc oxide	Poly I:C	B16–F10 mouse melanoma model	suppressed tumor cell growth	[28]
PS	Poly I:C	C57BL/6 mouse model	High IL6 production; *tnfa, il15, il18, mip3a, and ip10* mRNA upregulation	[164]
PLGA	Paclitaxel LPS	B16–F10 mouse melanoma model	Increased TIL levels and tumor regression	[33]
Pyridyl disulfide	Paclitaxel or CpG	B16–F10 mouse melanoma model	Slowed tumor growth, increased CD8^+^/CD4^+^ T cell ratio	[27]
Albumin	Paclitaxel	Phase I studies	Combination with IL-2, IFN-α, cisplatin and temozolomide was too toxic; combination with atezolizumab was well tolerated	[53, 54]
Liposome	DOX	Phase I study	Combination with IL-18 is safe and biologically active	[55]
PEG-liposome	DOX	Phase I study	Functional IL-6R blocking with tocilizumab is feasible and safe in combination with PEG-liposomal DOX	[56]

*DOX* doxorubicin, *HA* hyaluronic acid, *LPS* bacterial lipopolysaccharide, *PEG* polyethylene glycol, *PEI* polyethylenimine, *PLGA* poly(lactic-*co*-glycolic acid), *PPS* poly(propylene sulphide), *PS* polysaccharide, *TIL* tumor infiltrating lymphocytes

^a^Compared to free soluble agent, when applicable

Concerning clinical work, several nanoparticles encapsulating chemotherapeutic drugs have been approved by FDA or are currently being tested in clinical trials for various types of malignancies; these include: liposomal doxorubicin [35–38], daunorubicin [39–43], irinotecan [44], vincristine [45–48] and albumin-bound paclitaxel (*nab*-paclitaxel) [49–52]. Despite this, clinical studies in combination with immunotherapy regimens are only slowly starting to emerge. One recent Phase I study investigated the combination of *nab*-paclitaxel with immunotherapy (co-administration of soluble IL-2 and IFN-α) in metastatic melanoma, but the study failed to identify the maximum tolerated dose due to recorded toxicity at the lowest concentration tested and also the limited number of patients enrolled (10) [53]. On the other hand, a Phase Ib study in metastatic triple-negative breast cancer patients confirmed the safety and the therapeutic benefit of a combination of a checkpoint inhibitor (anti-PD-L1: atezolizumab) with *nab*-paclitaxel, setting the basis for an ongoing Phase III clinical trial [54]. Finally, another Phase I study in recurrent ovarian cancer provided evidence for safety and biological activity of pegylated liposomal doxorubicin in combination with interleukin-18 [55]; similar positive outcomes were also reported for a combination of liposomal doxorubicin, anti-IL6-receptor antibody and IFN-α [56].

In light of these studies, it is clear that further work will be needed in the future to establish what the best encapsulation and administration strategies are (e.g. co-encapsulation and co-delivery *versus* sequential administration) as well as to identify the best drug combinations. To help the clinical translation the different formulations should also be tested in more sophisticated systems such as immunocompetent and/or humanized mouse models [57]. Finally, nanotherapies previously tested in humans (i.e. *nab*-paclitaxel) should be further investigated in combination with immunostimulatory agents (e.g. interleukins, checkpoint inhibitors, etc.) with and/or without encapsulation to potentially improve therapeutic outcomes [58].

## Adoptive T cell therapy

Adoptive T cell therapy (ACT) consists of the isolation of autologous tumor specific T cells from the patient’s peripheral blood or tumor biopsies, followed by ex vivo expansion and patient re-infusion to elicit an anti-cancer immune response [59]. Alternatively (especially for those type of cancers where cancer-specific T cells are less spontaneously occurring), T cells can be expanded from patient-genetically modified T cells expressing a tumor-specific T Cell Receptor (TCR) or a chimeric TCR composed of a synthetic antigen-binding Ig domain fused with TCR signalling components, called CAR receptor [60]. Despite promising results yielded in clinical trials for melanoma [61–63] and other cancer types [64–66], ACT still suffers from important drawbacks and challenges that limit its widespread use. Some of the major limitations include: (1) the time-consuming and costly procedure of ex vivo cell expansion which requires 5–6 weeks and specific equipment (e.g. bioreactors), (2) T cell persistence and functionality after infusion which usually necessitate administration of survival factors, and (3) systemic toxicity. Bioengineering approaches have recently tried to solve those issues by employing biomaterials in different ways. One successful strategy developed by Irvine and colleagues (so far in mouse models) is to conjugate nanoparticles loaded either with stimulating factors (IL-15 and IL-21) [67] or an immunosuppression-blocking drug (NSC-87877) [68] directly on the surface of expanded T cells, prior to infusion. Interestingly, this strategy enabled the local delivery of immunomodulatory agents at high concentration that sustained T cell proliferation and effector function with greatly increased therapeutic advantages and minimized toxic effects compared to systemic infusion [67, 68]. In a follow-up study, nanoparticles were decorated with T cell targeting antibodies and used to stimulate ACT cells in vivo instead of ex vivo prior to infusion [69]. This approach has the advantage of enabling multiple rounds of stimulation by repeated nanoparticle injections rather than a single stimulation step ex vivo. In particular, T cell targeting was achieved using either an ACT-T cell specific surface antigen (Thy1.1) to restrict targeting only to ACT cells, or IL-2 which would target less specifically the whole T cell compartment but with the advantage of providing also a stimulating signal. Results showed successful targeting efficiency of ACT cells with low binding to endogenous T cells in both cases; however IL-2-loaded nanoparticles were also able to induce repeated waves of ACT T cell expansion in tumor-bearing mice upon multiple injections, thanks to IL-2 signalling. Based on this proof-of-concept, current work is focusing now on loading drugs and immunomodulatory molecules on these T-cell targeting nanoparticles to further improve ACT therapeutic efficacy [69].

Concerning CAR T cells, a very recent breakthrough study explored the possibility to programme T cell in situ with the injection of DNA-carrying nanoparticles [70]. In particular, these nanoparticles were coated with anti-CD3 antibodies to target the T cell compartment and loaded with DNA encoding for a leukaemia-specific CAR T cell receptor. Tests in an immunocompetent leukaemia murine model showed correct T cell transduction and proliferation, leading to disease regression with an efficacy comparable to conventional adoptive CAR T cell therapy as well as reporting general safety without any systemic toxicity [70]. Such an approach is very promising since it circumvents the need to isolate and manipulate T cells ex vivo, an aspect linked with the major hurdles of current ACTs (see above) and it should be therefore further investigated in the future for other cancer types as well as considered for clinic translation. Another explored route to improve current ACTs is the employment of artificial antigen presenting cells (aAPCs) to stimulate T cell expansion. To provide appropriate signalling, aAPCs must present on their surface a peptide-MHC complex that binds to the TCR (signal 1) and a CD28 antibody to provide co-stimulatory signalling (signal 2); in addition they could also provide adjuvants such as IL-2, IL-15 or IL-21 to further sustain T cell expansion (signal 3) [71]. aAPCs offer the advantage of avoiding the need to generate patient-specific DCs to stimulate tumor-specific T cells either ex vivo or in vivo as well as providing a versatile and cost-effective platform for T cell stimulation and expansion. On the other hand, a major disadvantage is the surface rigidity that fails to recapitulate the dynamic changes of the APC surface upon T-cell interaction. Important breakthroughs have been made recently in this field, thanks to the employment of biomaterials, substantially contributing to improve aAPC efficacy. Initial studies demonstrated that polymer-based nanoparticles were much less efficient than microparticles in inducing in vitro T cell functional responses (with notably no proliferation) suggesting that micron-sized beads, which are close in size to T cells, provide optimal T-cell stimulation [72]. However, Perica et al. recently reported a nano-sized aAPC platform based on either iron-dextran paramagnetic nanoparticles or quantum dot nanocrystals both able to induce antigen specific-T cell proliferation and tumor shrinkage in a melanoma mouse model [73]. This discovery constitutes a critical improvement for aAPCs in vivo applications since, contrary to micro-sized particles, nano-sized ones are able to passively drain to lymph nodes [74] where they could gain access to a large pool of T cells to prime, making them more suitable and efficient for in vivo administration. The same group has also recently developed aAPC magnetic nanoparticles conjugated to CD28-antibody and MHC-I-tumor antigen complexes as a strategy to isolate tumor-specific T cells from peripheral blood using magnetic columns, followed by ex vivo expansion [75]. The enrichment step was used to remove unspecific T cells that would compete with tumor-specific T cells for growth factors and decisively improved the antigen-specific cell fold expansion both in vitro and in vivo after transfer. Other important improvements came from using ellipsoidal micro-particles instead of spherical ones in order to decrease the surface curvature and therefore increase the area available for T-cell contact [76] highlighting the importance of not only the stimulating signals, but also the geometry and design of aAPCs to provide a successful stimulation. In light of this, it will be important in the future to also explore alternative geometries to mimic for example membrane protrusions or lamellipodia that are involved in T cell-APC interactions [77], in an attempt that will stimulate both the cancer immunotherapy and the bioengineering fields providing future synthetic challenges [78]. Finally, while up to now aAPCs have been prepared by randomly distributing ligands on their surface, recent studies suggest that the juxtaposition and the relative positions of signal 1 and 2, as well as their surface density [79, 80], are also important to efficiently stimulate T cells [78]. For example, using planar arrays it was shown that the presence of anti-CD28 at the periphery of the T cell contact site increased IL-2 secretion by CD4 T cells compared with having these signals combined in the center of the synapse [81]. The need to precisely control the pattern and distribution of ligands constitutes therefore another challenge for future bioengineering synthetic approaches.

## Cancer vaccines

Therapeutic cancer vaccines consist of using cancer antigens to pulse dendritic cells either in vivo or ex vivo followed by administration to patients to induce a cancer-specific immune response. These vaccines are therapeutic rather than preventive, since they are designed to treat a disease, which is already in course. The first attempts in this sense were injections of autologous tumor cells or tumor specific proteins administered alone or with an adjuvant [82–84], while more recently an alternative strategy has been developed by stimulating directly dendritic cells ex vivo with tumor associated or specific antigens (TAAs, TSAs) or whole tumor lysate (WTL) which are then re-infused into patients; this with the advantage of manipulating DCs during pulsing and activation to further improve their immunogenicity [85]. To this aim, dendritic cells can be obtained ex vivo by isolating monocyte precursors from peripheral blood followed by incubation with specific growth factors and cytokines such as GM-CSF, IL-4, IL-3, Flt3 ligand and c-Kit [86]. A great limitation of using TAAs is that the antigen(s) used has to be first identified and characterized which is not always possible for all types of cancers and it often requires extensive procedures. Moreover, there is also the possibility of immune escape by antigen loss from cancer cells [87]. Alternatively, DCs have also been pulsed with autologous WTL obtained from patient’s cancer cells by irradiation or cycles of freezing and thawing with the advantage of using a much larger pool of potential antigens and also avoiding the need for antigen identification [88–91]. Our group recently reported that HOCl oxidation of WTL prior to DCs ex vivo pulsing and maturation increased the uptake and presentation as well as improving the therapeutic outcome in an ovarian Phase I clinical trial [92, 93]. Another approach to increase immunogenicity of the lysate is to use heat, allowing increased production of heat shock proteins that further activate the immune response. This approach was tested in a pancreatic cancer mouse model with promising results [94]. Nonetheless, generating and activating DCs ex vivo is a time-consuming and costly procedure that can be potentially overcome using biomaterial vectors to deliver antigen(s) in situ. In recent years bio- and synthetic materials such as hydrogels, liposomes, matrices and nanogels which have the common feature of being biocompatible and non-toxic have been tested for the delivery of tumor antigen(s) in micro and nanoparticles in a great variety of combinations of different building blocks, antigens, adjuvants and targeting molecules (Table 2) [95]. Among these, due to their high biocompatibility and easy approval, liposomes have been largely explored and have also been tested in the clinic. Unfortunately, while certain formulations have shown discrete success in Phase I [96–100] and II trials [101, 102] showing good tolerance and survival improvement, Phase III trials have been less successful reporting limited benefits (BLP25 [103]) or failed to meet study endpoints (Allovectin-7 [104], product discontinued; Table 2). A major drawback of liposomes is their very short half life in the body and rapid clearance that limits the time frame in which they are active, a feature that could well be at the base of their reported failures [105]. A possible solution to this problem could be potentially offered by the implementation of Poly(lactic-*co*-glycolic acid) or PLGA in nanovaccine formulations. PLGA offers the advantage of being itself an immunostimulating agent, contributing therefore to the overall immune stimulation process rather that just acting as an inert carrier as well as being characterized by longer persistence in the human body and slow cargo release [105]. Several types of antigens such as proteins (e.g. ovalbumin (OVA) [106, 107], peptides (e.g. Hgp100_25–33_; TRP2_180–188_) [108, 109] and WTLs [110–113] have been encapsulated in PLGA nanoparticles and tested in in vitro systems and/or in mouse models showing positive outcomes in terms of efficient antigen delivery and elicited tumor-specific T cell responses. However none of these different formulations have been tested in humans yet. Another biopolymer tested in the clinic for cancer vaccine delivery is cholesteryl pullulan. Phase I trials in esophageal [114] or HER2-expressing [115, 116] cancer patients were carried out delivering well established cancer antigens (NY-ESO-1 protein and HER2 fragment, respectively) reporting good tolerance and the occurrence of antigen specific immune responses, while no Phase II or III trials appeared so far in the literature to our knowledge. Among other materials, chitosan also showed promising results for future translational applications. Chitosan is a cationic polysaccharide able to elicit an adjuvant innate immune response, like PLGA, further triggering DCs maturation. A recent study showed for example that subcutaneous injections of these NPs loaded with WTL in mice induced a specific cytotoxic T cell (CTL) response and reduced tumor size compared to control groups [117]. In an attempt to further improve particle uptake, DC-targeting and DC-maturation, several studies have used nano- or microparticles coated with DC-targeting ligands such as anti-CD40 [106, 118], anti-DEC-205 [106, 119, 120], anti-SIGN [121, 122], carbohydrates [107, 122] and/or TLR agonists [112, 123, 124] (Table 2). Collectively, results from all these studies confirmed the previous assumption that particle coating (or encapsulation in the case of TLR agonists) indeed improves DC maturation, antigen internalization and presentation, inducing a stronger immune response compared to non-targeted nanovaccines or free antigen(s) in mouse model systems. Few comparative studies were also able to identify better formulations over others (e.g. uptake of SIGN-antibody coated-nanoparticles was more efficient that carbohydrates-coated ones [122]; or, in another study, coating with CD-40 ligand was superior to DEC-205 or CD11c in terms of uptake [106]), even though a systematic classification and comparison is still lacking.

**Table 2:** List of recent studies investigating nanoparticle-mediated delivery of tumor antigen(s) either alone or in combination with adjuvant(s)/DC-targeting moieties for cancer therapeutic vaccination

Carrier	Loaded with	Study type	Outcome^a^	References
Liposome	Hsp70 peptide complex	Breast cancer mouse model	Enhanced immune response	[165]
Liposome	MUC1 peptide, TLR4 ligand	Phase I–II–III studies	Phase I studies: vaccine was well tolerated; phase II study in NSCLC: survival improvement; Phase III study in NSCLC: only improvement observed was in concurrent chemoradiotherapy with a 10.2 month improvement in median survival	[96, 97, 101, 103]
Liposome	HLA-B7 and β2-microglobulin DNA	Phase II-III studies	Phase II study in metastatic melanoma had a positive outcome, but phase III study failed and product is currently discontinued	[102, 104]
Liposome	NY-ESO-1, MAGE-A3, tyrosinase and TPTE RNA	Phase I study	Positive outcome in all 3 patients tested. Recruitment of more patients is currently undergoing	[98]
Liposome	Mix of different peptides	Phase I study	Phase I trial positive outcome, with induced de novo and specific T cell response	[99, 100]
Liposome	SOCS1, A20 siRNA	Mouse lymphoma model	Drastic enhancement in cytokine production resulting in significant tumor suppression	[166]
Liposome	E7 HPV	TC-1 lung mouse model	Induced specific CD8^+^T cell response and T_reg_ inhibition	[167]
Liposome	OVA, TLR3/9 ligands	C57BL/6 mouse model	Increased CD8^+^ T cell response	[123]
γ-PGA/Polylysine	Empty or ovalbumin	C57BL/6 mouse model	Comparative study: PGA has intrinsic immunogenic properties and induced a stronger immune response than polylysine when both loaded with ovalbumin	[160]
γ-PGA	Ovalbumin	C57BL/6 mouse model	γ-PGA immunogenic properties are TLR4 signalling-dependent	[168]
Cationic polymers (PE/C32)	CD40 ligand DNA, CpG + poly(I:C)	B16-F10 melanoma mouse model	Comparative study: C32 polimer was superior to PE. TLR ligands had a synergistic effect in triggering immune response	[124]
PLGA	WTL	In vitro	Co-culture of patient TILs with patient DCs pulsed with autologous WTL-NPs resulted in higher IFN-γ and lower IL-10 production compared to soluble WTL	[110, 111]
PLGA	WTL, CpG, polyI:C	TRAMP mouse model	Induced CTL response and tumor shrinkage	[112]
PLGA	WTL	In vitro	Increased T cell proliferation	[113]
PLGA	Ovalbumin TLR3/7 ligands; CD40, CD11c, or DEC-205 ab	C57BL/6 mouse model	NP coating with targeting molecules (CD40, CD11c or DEC-205 antibodies) induced a stronger immune response	[106]
PLGA	Ovalbumin, mannose	C57BL/6 mouse model	Decoration of ovalbumin-NPs with mannose moieties increased the efficiency of ovalbumin-specific CD4^+^ and CD8^+^ T cell responses	[107]
PLGA	TRP2_180–188_; TLR-4 ligand	B16-F10 melanoma mouse model	Immune stimulation in the tumor microenvironment, induction of antigen-specific CD8^+^ response	[108]
PLGA	Hgp100_25–33_ TRP2_180–188_	C57BL/6 mouse model	Increased antigen-specific T cell response	[109]
Cholesteryl pullulan	HER2 fragment; NY-ESO-1 protein	Phase I studies	Vaccine was well tolerated and induced antigen-specific immune responses	[114–116]
Chitosan	Ovalbumin, alginate	In vitro	Sugar-coated NP induced higher IFN-γ production in co-culture assays	[169]
Chitosan	WTL, mannose	B16 melanoma mouse model	Increased tumor growth inhibition	[117]
BSA/pyridine	Ovalbumin	In vitro	This type of nanogel had intrinsic adjuvant properties	[170]
Nanogel	Ovalbumin, galactose	B16-OVA mouse model	(pH-sensitive system) cytosolic antigen release; ROS production and increased MHC-I antigen presentation	[133]

*γ-PGA* poly(γ-glutamic acid), *BSA* bovine serum albumin, *NP* nanoparticle, *NSCLC* non-small-cell carcinoma, *PLGA* poly(lactic-*co*-glycolic acid), *TLR* toll-like receptor, *TRAMP* transgenic adenocarcinoma of the mouse prostate, *WTL* whole tumor lysate

^a^Compared to free soluble agent, when applicable

Another direction in which nanovaccine research has recently focused on is the development of pH-sensitive nanoparticles. These nanoparticles, once internalized, are able to disrupt endosomes leading to antigen(s) release in the cytosol, a process known to promote cross presentation by DCs and enhance CTL over humoral response [125]. This approach has been successfully attempted with different biomaterials including liposomes [126–128], hydrogels [129], micelles [130, 131] and synthetic polymers [132]. Overall, all these studies used nano-assisted delivery of OVA in mice as a model system and showed positive results including increased MHC-I antigen presentation and induction of OVA-specific CD8^+^ T cell response. Furthermore, a recent study using a pH-sensitive galactosyl dextran-retinal (GDR) nanogel for OVA encapsulation was able to show that the lysosome rupture triggered by nanoparticles could directly induce reactive oxygen species (ROS) production in DCs, augmenting proteasome activity and downstream MHC I antigen presentation [133]. These interesting results suggest therefore that pH-sensitive nanocarriers constitute a very promising scaffold for future translational work.

In conclusion, a great variety of scaffolds, materials and antigens have been tested for cancer vaccine delivery alone or in combination with specific surface receptors, and adjuvants that can improve DC-targeting and maturation. Despite these efforts achieved important results, further comparative studies are needed in order to understand which are the most promising and suitable biomaterials and to identify the best combinations of antigen(s), adjuvants and targeting molecules to obtain the best immune response. Enhancement of cross presentation by cytosol localization of the antigen(s) plays also a significant role in terms of CD8^+^ T cell polarization and should be studied and exploited in-depth in the future. Finally, tests in more complex systems that better represent human settings (e.g. humanized mouse models) [57] and for the delivery of epitopes more clinically relevant (e.g. other than OVA) or more immunogenic (e.g. oxidized WTL [92, 93] or heated lysate [94]) will help in translating these strategies into the clinic as well as potentially achieving better therapeutic outcomes.

## Circulating tumor cells isolation and detection

Circulating tumor cells (CTCs) are cancer cells that shed from the tumor primary site and after entering the bloodstream extravasate and arrest at a second distal site to initiate cancer metastasis [134]. Despite their first report dates back to 1869 [135], a great amount of interest towards CTCs and their use as predictive biomarkers for cancer metastasis has only emerged in the last two decades. This is mainly due to the technical challenges linked with detecting and isolating very rare cells (usually one in 10^6^–10^9^ hematologic cells [136]) which are also often highly heterogenic [137–139]. Several bioengineering solutions have been recently developed addressing these issues. One common strategy employs magnetic nanoparticle coated with specific ligands targeting CTCs (e.g. anti-EpCAM) that enables CTC separation and enrichment from blood samples by simply applying a magnetic field [136]. Other isolation techniques rely on Au nanoparticles, quantum dots, graphene or dendrimers coated with different CTC-targeting moieties such as lectins, tumor antigens or aptamers and have already been extensively reviewed elsewhere [140–142]. Despite great advances in biomaterial formulations for the detection and isolation of CTCs, their therapeutic implications have been largely unexplored yet, especially in the immunotherapy field. CTCs can be in fact isolated with a “simple” blood test (often referred to as liquid biopsy), contrary to solid tumors which require invasive surgery, and constitute a precious tool to assess genotypic and phenotypic features at a personalized level [143]. For example, CTCs genotyping and phenotyping could be potentially used to inform cancer vaccination strategies permitting the identification in real time of present antigens or, on the opposite, of antigen-loss due to selective pressure. On the other hand, isolated CTCs could constitute also a potential source of antigens to pulse autologous dendritic cells for personalized cancer vaccine formulations. Analogous strategies have been recently applied to instruct chemotherapeutic regimens such as HER2-receptor antagonists in breast cancer patients. Surprisingly, in several cases HER2 was detected in CTCs in metastatic patients that were previously negative at original diagnosis at the primary tumor site [144–146] and in one particular study three over four of these patients treated with anti-HER2 therapy (trastuzumab) showed evidence of complete or partial response [145]. These examples, besides demonstrating the heterogeneity and the dynamic nature of cancer, illustrate also the critical role that CTCs could play in guiding therapeutic efforts [147]. Thus, we envisage that in the future new studies will appear linking CTCs analysis and detection with immunotherapy. However, the success of these future approaches will rely in the high yield isolation of CTCs in a viable form. To this aim, several proof-of-concept studies showed the possibility to isolate CTCs from leukapheresis products, in order to screen blood volumes much larger (~10 L) than the commonly used for CTCs analysis (5–10 mL) [148–150]. Alternatively, other groups are developing implantable scaffolds that are able to capture and trap CTCs which could be subsequently recovered and analyzed [151, 152]. In addition to this, the material could also be seeded with cells, or adjuvants to modulate the immune environment within the scaffold [152]. Ongoing work is focusing in further developing these proof-of-concept studies towards translational applications. It should also be noted that developments in CTCs sequestering and elimination will be immensely powerful in fighting cancer, considering that 90% of cancer mortality is caused by metastasis [153]; hence efforts in this direction could be potentially extremely rewarding.

## Route of administration

One of the crucial aspects for a successful nanotherapy is the route of administration which should ensure both targeted delivery of the regimen at its active site (this being for example the tumor site or the lymph nodes) combined with as few as possible collateral effects and invasiveness. Regarding those formulations that target the tumor site, several studies applied intratumoral or peritumoral injection of nanoparticles loaded with immunostimulatory molecules (such as: IL-12 [154], IL-15 superagonist [155], IL2 and TGF-β [19] among others) with positive outcomes, reporting the initiation of an immune response in tumor-bearing mice. Interestingly, one particular study demonstrated how intratumoral injection of liposomes carring anti-CD137 and IL-2 enabled an otherwise lethal treatment (compared to soluble anti-CD137 and IL-2) [156]. Although intratumoral injection ensures high local drug concentration and targeted delivery, a lot of studies apply more straightforward intravenous or subcutaneous injections and exploit instead the above mentioned EPR effect to passively accumulate the cargo at the tumor site. However, raising evidence suggests that the EPR effect works in rodents but not in humans (probably due to the large differences in tumor-to-body weight ratio and differences in the tumor microenvironment, between murine models and human cancers) [9], a fact that should be taken into careful consideration for clinical translation. In particular, this issue could potentially be solved by coating the surface of nanocarriers with ligands targeting receptors overexpressed by cancer cells (e.g. transferrin, folate, epidermal growth factor or glycoproteins receptors [157]) allowing therefore a more focused and active targeting.

Regarding formulations that target instead lymph nodes (e.g. cancer vaccines), nanocarriers can be administered either parentally (intramuscularly or subcutaneously, as in the majority of the studies), or intranodally. In the former case, the size of the nanoparticle is crucial in determining the mechanism of trafficking to the lymph nodes. In fact, while smaller particles (<200 nm) are able to passively drain through lymphatic system to finally reach the lymph nodes, larger particles cannot and have to be first engulfed by peripheral DCs which then migrate from the injection site to the lymph nodes [74]. On the other hand, the intranodal injection, although more technically challenging and invasive, ensures direct delivery and accumulation at the lymph node enabling the use of also microparticles which, contrary to nanoparticles, are able to persist longer at the lymph node releasing their cargo in a more prolonged and sustained fashion [158].

Finally, in an effort to balance improved targeted delivery versus limited invasiveness, a recent study pioneered the use of microneedle patches (MNs) to deliver antibodies against the checkpoint inhibitors PD1 and CTL4 in a melanoma mouse model. Results showed that MNs can painlessly pierce the mouse skin and efficiently deliver their cargo to regional lymph and capillary vessels ensuring disease control in 70% of mice over 2 months (end time point) [159]. This promising proof-of-concept study shows therefore that MNs could efficiently combine target delivery with easy and non-invasive administration, holding great potential for delivery of also other immunotherapeutic regimens in the future.

## Conclusion and future perspectives

As highlighted by the sheer amount of studies reviewed here, nanoparticle delivery systems are a very versatile platform to address crucial limitations of current cancer immunotherapy, both in vivo and ex vivo. In particular, nanotechnology and bioengineering approaches have greatly enhanced the efficacy of immunotherapies by ensuring targeted delivery, limited systemic toxicity, and increased local concentrations of therapeutic regimens. Despite many advances, a great deal of work is still needed in the future to further characterize and optimize the various platforms. First of all, comparative studies are importantly required to identify what are the most advantageous materials (e.g. liposomes versus synthetic polymers etc.), sizes, compositions and other biophysical aspects, for each application. Few of this type of studies already appeared in the literature [74, 124, 160] but a systematic classification is still lacking. Furthermore, comparative studies aimed at identifying the best synergistic combinations of immunomodulatory molecules (e.g. cytokines, chemotherapeutic agents, antigens etc.), coadjuvantes (e.g. TLR receptor ligands) and/or target moieties (e.g. DC or T cell specific antibodies) will also help to progress the future of these therapies. Another key aspect to further investigate is the route of administration, in order to guarantee efficient delivery while limiting the treatment invasiveness. In this sense, a recent breakthrough study reported the successful use of MNs for the delivery of checkpoint inhibitors [159], a route of administration that should be further tested for the release of also different nanotherapies. Finally, apart from few cases, the majority of these formulations have not been implemented yet in the clinic. To this aim, studies in more sophisticated models such as “humanized” mouse models [57, 161] that better recapitulate the human settings of the disease will be key to support and boost future clinical translations. In conclusion, biomaterials constitute a powerful tool to overcome challenges with current immunotherapies, however we may have just started scratching the surface of the future bioengineered solutions for cancer immunotherapy.

## Abbreviations

ACT: adoptive T cell therapy
APC: antigen presenting cell
aAPC: artificial antigen presenting cell
CTC: circulating tumor cell
CTL: cytotoxic T lymphocyte
DC: dendritic cell
EPR: enhanced permeability and retention effect
GDR: galactosyl dextran-retinal
ICD: immunogenic cell death
TIL: infiltrated lymphocyte
MN: microneedle
PLGA: poly(lactic-*co*-glycolic acid)
ROS: reactive oxygen species
TCR: T cell receptor
TLR: toll-like receptor
TAA: tumor associated antigen
WTL: whole tumor lysate
